# Impact of parents’ need for care on middle-aged women’s lifestyle and psychological distress: evidence from a nationwide longitudinal survey in Japan

**DOI:** 10.1186/s12955-018-0890-2

**Published:** 2018-04-13

**Authors:** Takashi Oshio, Mari Kan

**Affiliations:** 1 Institute of Economic Research, Hitotsubashi University, 2-1 Naka, Kunitachi, Tokyo, 186-8603 Japan; 2 School of Economics, University of Hyogo, 8-2-1 Gakuen-Nishi-machi, Nishi-ku, Kobe, Hyogo 651-2197 Japan

**Keywords:** Informal caregiving, Mental health, Lifestyle, Japan

## Abstract

**Background:**

Many studies have separately addressed the associations of informal caregiving with coresidence, a caregiver’s work status, and health conditions, but not jointly. We examined how their parents’ need for care affects middle-aged women’s lifestyle and psychological distress, considering the potential simultaneity of decisions on caregiving and living adjustments.

**Methods:**

We used 22,305 observations of 7037 female participants (aged 54–67 years) from a nationwide longitudinal survey in Japan conducted during 2009 and 2013. We considered the occurrence of parents’ need for care (OPNC) as an external event and estimated regression models to explain how it affected the probabilities of the participants becoming caregivers, coresiding with parents, and working outside the home. We further conducted the mediation analysis to examine how the impact of OPNC on participants’ psychological distress measured by Kessler 6 (K6) scores was mediated by caregiving and living adjustments.

**Results:**

OPNC made 30.9% and 30.3% of middle-aged women begin informal caregiving for parents and parents-in-law, respectively, whereas the impact on residential arrangement with parents or work status was non-significant or rather limited. OPNC raised middle-aged women’ K6 scores (range: 0–24) by 0.368 (SE: 0.061) and 0.465 (SE: 0.073) for parents and parents-in-law, respectively, and informal caregiving mediated those impacts by 37.7% (95% CI: 15.6–68.2%) and 44.0% (95% CI: 22.2–75.4%), respectively. By contrast, the mediating effect of residential arrangement with parents or work status was non-significant.

**Conclusions:**

Results underscore the fact that OPNC tends to promote middle-aged women to begin informal caregiving and worsen their psychological distress.

## Background

Informal caregivers provide most of the long-term care in many countries. Owing to longer life expectancy and a lower number of siblings, we now face a higher probability of individuals having to provide informal care to old parents [1]. Hence, the occurrence of parents’ need for care (OPNC) is a key driver of the change in the lifestyle of middle-aged individuals, especially women, who still tend to play a dominant role in informal care. If their parents happen to need care, adult children are probably forced to consider who will provide care to them, whether they will start coresiding with parents [2–5], whether a caregiver will stop work outside the home [6, 7], and so on.

Actually, many studies have already addressed the associations of informal caregiving with coresidence, a caregiver’s work status, and health conditions, albeit not jointly. Poor health of parents tends to raise the probability of coresidence with their adult children [2–5]. In comparison, mixed findings have been reported on the association between informal caregiving and a caregiver’s work status. However, many studies have shown that the effect of informal caregiving on employment is relatively limited [6, 7]. One possible reason is the endogenous selection for assuming a caregiving role. Specifically, women, who tend to have a weaker attachment to the labor market, are more likely to take on the caregiving role [8].

A key limitation of previous studies is that they have often considered informal caregiving as an exogenous variable, thereby ignoring possible simultaneity biases. Further, most of these studies did not consider the simultaneity of decisions on informal caregiving and other behaviors, such as coresidence with parents and work outside the home, which are likely to interact with each other.

In the present study, we attempted to control for potential biases owing to endogeneity of informal caregiving and simultaneity of decisions on informal caregiving and other behaviors, in order to examine the relevance of informal caregiving to the life arrangements and well-being of middle-aged women more precisely. Therefore, we focused on OPNC, which was considered largely exogenous, and examined how the middle-aged women responded to it in terms of caregiving, residential relationship with parents, and work status, taking into account the impact of their pre-OPNC statuses as well as their interactions under the framework of a simultaneous regression model.

We further examined how the onset of caregiving and living adjustments mediated the impact of OPNC on the middle-aged women’s psychological distress, based on the theoretical framework of the mediation analysis [9, 10]. It is reasonable to predict that these living adjustments, which are likely correlated with each other, will affect middle-aged women’s psychological distress, especially if they become caregivers [11, 12]. Indeed, studies have evidenced that informal caregiving has a negative association with a caregiver’s health and quality of life [13–15]. However, some studies suggest that conditions surrounding caregiving—such as coresidence with parents and employment status—tend to mediate the impact of informal caregiving on a caregiver’s psychological distress [16–20]. In this study, we computed the mediating effects along with their statistical significance of caregiving and living adjustments.

## Methods

### Study sample

We used longitudinal data obtained from a nationwide, population-based longitudinal survey titled, “The Longitudinal Survey of Middle-Aged and Older Adults,” conducted by the Japanese Ministry of Health, Labour and Welfare (MHLW). Samples in the first wave were collected nationwide from individuals between the ages of 50–59 years in November 2005 through a two-stage random-sampling procedure. First, 2515 districts were randomly selected from 5280 districts used in the MHLW’s nationwide, population-based “Comprehensive Survey of the Living Conditions of People on Health and Welfare,” which was conducted in 2004. The 5280 districts, in turn, had been randomly selected from about 940,000 national census districts. Second, depending on the population size of each district, 40,877 residents aged 50–59 years as of October 30, 2005 were randomly selected.A total of 34,240 individuals responded (response rate: 83.8%). The second to ninth waves were conducted in early November of each year from 2006 to 2013, with no additional sampling (average attrition rate in each wave: 4.3%).

We took full advantage of the longitudinal structure of the dataset to capture the timing of OPNC and the middle-aged women’s responses to it in terms of caregiving, residential relationship with parents, and work status, taking into account the impact of their pre-OPNC statuses. Specifically, we first compiled the data on female participants from the fourth to ninth waves (2008–13), because information on each individual’s parents’ need for care was collected only from the fourth wave. We then limited our analysis to the data of female participants who had not faced parents’ need for care—and thus had not provided caregiving to parents—in the year prior to the survey year. It means that we excluded the data of participants who had already faced parents’ need for care in the fourth wave (2008) and focused on the participants’ data from the fifth wave (2009) onwards. This allowed us to capture the exogenous impact of OPNC in the survey year. We also excluded the data of participants whose parents died in the survey year. After further excluding participants with missing data, we used 22,305 observations of 7303 women for the statistical analysis, in which we focused on their responses to OPNC at the survey year.

### Measures

The survey asked respondents whether care was needed for each family member. We collected the data of parents’ need for care and constructed a binary variable in which the emergence of parents’ need for care in the survey year (after no need was reported in the previous year) was scored as “1” and other conditions were scored as “0,” for the participants’ parents (father and/or mother) and parents-in-law (father-in-law and/or mother-in-law). Similarly, we constructed binary variables for participants’ provision of informal care to and coresidence with parents and parents-in-law, by allocating “1” if the participant was providing informal care and was residing with parents or parents-in-law, and “0” otherwise. We also constructed a binary variable for work outside the home by allocating “1” if the participant answered that she was engaged in any paid job and “0” otherwise.

Additionally, we focused on the impact of OPNC on women’s psychological distress measured by K6 scores [21, 22]. The reliability and validity of K6 scores have been demonstrated in a Japanese population [22, 23]. Participants were asked to complete a six-item psychological distress questionnaire: “During the past 30 days, about how often did you feel a) nervous, b) hopeless, c) restless or fidgety, d) so depressed that nothing could cheer you up, e) that everything was an effort, or f) worthless?” Responses were rated on a 5-point scale (1 = none of the time to 5 = all of the time). K6 score (range: 0–24) was constructed by subtracting six from the sum of the responses. Higher K6 scores reflect higher levels of psychological distress. We additionally focused on the proportion of respondents with K6 score ≥ 5, which has been found to indicate mood or anxiety disorders in a Japanese population [24]. For the entire respondents in this study sample (*N* = 22,307), Cronbach alpha coefficient for K6 scores was 0.90, K6 scores’ mean and standard deviation were 3.4 and 4.0, respectively, and the proportion of those with K6 score ≥ 5 was 30.0%.

As for control variables, we used the respondent’s age, educational attainment (junior high school, high school, college or above, other), having a spouse, and household expenditure as a proxy of household income. These factors were taken into account, because they were expected to affect the costs—both pecuniary and psychological—of informal caregiving and living arrangements, and correspondingly, their impact on psychological distress. Household expenditure was adjusted for household size by dividing the reported value of household expenditure by the square root of the number of members in the household, as was done in recent publications of the Organisation for Economic Co-operation and Development [25].

### Analytic strategy

We compared the probabilities of three variables—becoming a caregiver, coresiding, and working outside the home—between those who faced parents’ need for care and those who did not, without controlling for other variables. However, as explained in the Introduction, we had to control for potential biases owing to the endogeneity of informal caregiving and simultaneity of decisions on informal caregiving, coresidence, and working outside the home as well as their statuses prior to the survey year. Therefore, we jointly estimated a set of linear regression models within the framework of the seemingly unrelated regression (SUR) model [26]:

$$ {\displaystyle \begin{array}{c}{\mathrm{Caregiving}}_t={\alpha}_1{\mathrm{OPNC}}_t+{\beta}_1{\mathrm{Coresidence}}_{t-1}+{\gamma}_1{\mathrm{Work}}_{t-1}+{\boldsymbol{Z}}_t{\boldsymbol{\updelta}}_1+{\varepsilon}_{1t}\\ {}{\mathrm{Coresidence}}_t={\alpha}_2{\mathrm{OPNC}}_t+{\beta}_2{\mathrm{Coresidence}}_{t-1}+{\gamma}_2{\mathrm{Work}}_{t-1}+{\boldsymbol{Z}}_t{\boldsymbol{\updelta}}_2+{\varepsilon}_{2t}\\ {}{\mathrm{Work}}_t={\alpha}_3{\mathrm{OPNC}}_t+{\beta}_3{\mathrm{Coresidence}}_{t-1}+{\gamma}_3{\mathrm{Work}}_{t-1}+{\boldsymbol{Z}}_t{\boldsymbol{\updelta}}_3+{\varepsilon}_{3t}.\end{array}} $$

Here, the subscript *t* indicates year *t* (*t* = 2009, 10, 11, 12, and 13), and ***Z*** and *ε*_*i*_ (*i* = 1, 2, and 3) indicate a set of control variables and an error term. We estimated *α*_*i*_ and *β*_*i*_, which are coefficients of OPNC and Coresidence, respectively, as well as ***δ***_*i*_, which is a set of coefficients of each control variable included in ***Z***. This set of regression models attempted to capture the impacts of OPNC on caregiving, coresidence, and work, assuming that these three variables were affected by coresidence and work in the previous year, and that the error terms were correlated with each other.

The focus was on the estimated value of *α*_*i*_, which indicates the impact of OPNC on caregiving, coresidence, and work. Because we limited the analysis to the respondents who did not face OPNC (and thus did not engage in caregiving) in the previous year, the estimated value of *α*_1_ indicates the probability of newly becoming a caregiver in response to OPNC. ***Z*** included age, educational attainment, having a spouse, and household expenditure, as mentioned earlier.

One may be tempted to estimate a multivariate probit model rather than a set of linear regression models within the framework of the SUR model, considering that three dependent variables are all binary ones. However, we did not use a multivariate probit model because “no OPNC” (OPNC = 0) perfectly predicted “no caregiving” (caregiving = 0) in the first caregiving model, thus omitting OPNC from regression. It has also been known that linear probability models obtain results generally similar to those of probit or logistic models and that their theoretical flaws can be disregarded in most cases [26].

We further estimated regression models to explain the extent to which OPNC affected K6 scores and how its impact was confounded by caregiving, coresidence, and work. Specifically, we first estimated the benchmark model (Model 1), which explained K6 scores by OPNC. Next, we estimated three models (Models 2–4), each of which included caregiving, coresidence, and work as an additional predictor. Then, we examined how the results were affected by adding all of these variables in Model 5. In all these models, we included a set of control variables (***Z***) as well as K6 scores, coresidence, and work status in the previous year.

Finally, we conducted the mediation analysis [9, 10] to examine how the impact of OPNC was mediated by three potential mediators: caregiving, coresidence, and work. Based on the results of (i) the SUR model (which examined the impacts of OPNC on each of three mediators) and (ii) Model 1 (which explained K6 scores by OPNC), and (iii) Model 5 (which explained K6 scores by OPNC and three mediators), we computed the mediating effects of each of three mediators. We examined their statistical significance by bootstrap estimating their 95% confidence intervals with 3000 replications.

## Results

### Descriptive analyses

Table 1 summarizes the key characteristics of 7037 participants at baseline (in 2009). Among the participants, 11.3%, and 22.0% were residing with parents and parents-in-law, respectively. We also observed that 63.5% of the participants were working outside the home.

**Table 1:** Key characteristics of 7037 participants at baseline (in 2009)

Educational attainment	Proportion (%)	
Junior high school		12.7
High school		64.1
Junior college		14.6
College or above		8.3
Having a spouse		88.0
Residing with parents		11.3
Residing with parents-in-law		22.0
Working outside home		63.5
Age (years)	*M*	58.0
	*SD*	(2.7)
Monthly household expenditure (thousand yen)^a^	*M*	230.0
	*SD*	(283.0)

^a^Adjusted for household size

Table 2 compares the probabilities of caregiving, coresidence, and work between women who faced OPNC and those who did not. It was found that 30.7% and 29.7% of the participants started caregiving in response to the OPNC of parents and parents-in-laws, respectively. The difference in the probabilities of caregiving in the right column of Table 3 indicates the probability of newly becoming a caregiver in response to OPNC, because the probability of caregiving was equal to zero among those who did not face OPNC. The probabilities of coresidence and work were lower among women who faced OPNC than those who did not, but their differences (ranging between 1.8–7.2%) were much more limited as compared to those with the probabilities of caregiving. Table 2 also shows that the mean K6 score and the proportion of those with a K6 score ≥ 5 was much higher among women who faced OPNC than among those who did not, for both parents and parents-in-law.

**Table 2:** Behavioral probabilities and K6 scores of women with and without parental need for care^a^

	The need of care		Difference (A-B)	
	Occurred (A)	Not occurred (B)		
Parents (*n* = 15,972)				
Caregiving	30.7%	0	30.7%	***
			*SD* (0.4%)	
Coresidence	13.7%	15.5%	−1.8%	*
			*SD* (0.8%)	
Work	55.1%	60.5%	−5.4%	***
			*SD* (1.0%)	
K6 score (range: 0–24)	*M* 3.79	3.20	0.59	***
	*SD* (0.08)	(0.03)	(0.00)	
K6 score ≥ 5	33.3%	28.4%	4.9%	***
Number of observations	2648	13,324		
Parents-in-law (*n* = 10,887)				
Caregiving	29.7%	0	29.7%	***
			*SD* (0.5%)	
Coresidence	33.1%	40.3%	−7.2%	***
			*SD* (1.2%)	
Work	55.7%	62.0%	−6.3%	***
			*SD* (1.2%)	
K6 score (range: 0–24)	*M* 3.90	3.20	0.70	***
	*SD* (0.10)	(0.04)	(0.10)	
K6 score ≥ 5	34.4%	29.3%	5.1%	***
Number of observations	2086	8801		

^a^All variables were evaluated in each wave when parents’ need for care emerged

^***^p < 0.001, ^*^p < 0.05

**Table 3:** Estimated impact of the occurrence of parents’ need for care on women’s behavior^a^

	Women’s behavior								
Explanatory variables	Caregiving			Coresidence			Work		
	Coef.		SE	Coef.		SE	Coef.		SE
Parents (*n* = 15,972)									
Occurrence of parents’ need for care	0.309	***	(0.004)	0.013	***	(0.003)	−0.011		(0.006)
Coresidence in the previous year	0.062	***	(0.004)	0.934	***	(0.003)	−0.001		(0.006)
Work in the previous year	−0.012	***	(0.003)	0.002		(0.002)	0.819	***	(0.005)
Parents-in-law (*n* = 10,887)									
Occurrence of the need for care	0.303	***	(0.005)	−0.006		(0.004)	−0.024	***	(0.007)
Coresidence in the previous year	0.087	***	(0.004)	0.928	***	(0.004)	0.011	*	(0.006)
Work in the previous year	0.003		(0.004)	0.008	*	(0.004)	0.813	***	(0.006)

^a^Adjusted for age, educational attainment, having a spouse, household expenditure in all models

^***^p < 0.001, ^*^p < 0.05

### Regression analyses

Table 3 summarizes the estimated impact of OPNC on women’s behavior. We observed that 30.9% and 30.3% of women started caregiving in response to the OPNC of parents and parents-in-law, respectively. The magnitude of this impact was almost the same as that observed in Table 2 (30.7% and 29.7%). Coresidence in the previous year raised the probability of caregiving for both parents (6.2%) and parents-in-law (8.7%), whereas work in the previous year slightly reduced it for parents. As for coresidence, OPNC slightly raised the probability of coresidence for parents (1.3%) and it had no significant impact (0.6%) for parents-in-law. Instead, residential status in the previous year was a key determinant of the current residential status. A negative impact of OPNC on work (1.1% for parents and 2.4% for parents-in-law) was rather limited and smaller than that suggested by the descriptive comparisons in Table 2. We further observed that previous work status strongly determined the current one.

Table 4 presents the estimation results of Models 1–5, which explain how OPNC affected women’s K6 score. As the benchmark model, Model 1 showed that OPNC raised women’s K6 scores by 0.368 and 0.465 for parents and parents-in-law, respectively. These impacts were equivalent to 0.09 and 0.13 standard deviation of K6 scores. Model 2 showed that the impact of OPNC was substantially mediated by becoming a caregiver for both types of parents. The inclusion of caregiving substantially attenuated the association between OPNC and K6 scores—the coefficient declined by 38.1% to 0.227 for parents and by 43.6% to 0.263 for parents-in-law (0.220)—while caregiving had a significant, positive correlation with K6 scores for both parents (0.454) and parents-in-law (0.668).

**Table 4:** Estimated impact of the occurrence of parents’ need for care on women’s K6 scores^a^

	Model 1			Model 2			Model 3			Model 4			Model 5		
	Coef.		SE	Coef.		SE	Coef.		SE	Coef.		SE	Coef.		SE
Parents (*n* = 15,972)															
Occurrence of parents’ need for care	0.368	***	(0.061)	0.227	***	(0.071)	0.366	***	(0.061)	0.367	***	(0.061)	0.228	***	(0.071)
Caregiving				0.454	***	(0.121)							0.449	***	(0.121)
Coresidence							0.119		(0.164)			(0.080)	0.072		(0.165)
Work										−0.062			−0.057		(0.080)
Parents-in-law (*n* = 10,887)															
Occurrence of parents’ need for care	0.465	***	(0.073)	0.263	**	(0.085)	0.465	***	(0.073)	0.460	***	(0.073)	0.255	**	(0.085)
Caregiving				0.668	***	(0.145)							0.674	***	(0.146)
Coresidence							−0.028		(0.158)				−0.109		(0.159)
Work										−0.211	*	(0.100)	−0.202	*	(0.100)

^a^ Adjusted for age, educational attainment, having a spouse, household expenditure, coresidence, and work in the previous year in all models

^***^p < 0.001, ^**^p < 0.01, ^*^p < 0.05

Models 3 and 4 showed that coresidence or work did not have any positive association with K6 scores, leaving the impact of OPNC virtually intact, for both parents and parents-in-law, while work reduced K6 scores in the case of caring for parents-in-law. Finally, Model 5, which included all related variables, largely mirrored the results in Models 2–4; the coefficients for OPNC and caregiving remained close to those in Model 2, while the coefficients for coresidence and work remained almost intact from Models 3 and 4, respectively.

Lastly, Table 5 presents the results of the mediation analysis, based on the results of the SUR models presented in Table 3 and those of Models 1 and 5 presented in Table 4. For caregiving to parents, OPNC raised K6 score by 0.368, and 37.7% of this impact (i.e., 0.139) was mediated by caregiving. In contrast, coresidence or work did not significantly mediate the impact of OPNC on K6 scores. We found similar results for parents-in-low; caregiving mediated 44.0% of the impact of OPNC K6 scores, while coresidence or work did not work as a mediator.

**Table 5:** Estimated impact of the occurrence of parents’ need for care on women’s K6 scores mediated by their behavior^a^

	Coefficient	95% CI^b^	Proportion (%)	95% CI^b^
Parents (*n* = 15,972)				
Mediated by:				
Caregiving	0.139	(0.058, 0.217)	37.7	(15.6, 68.2)
Coresidence	0.001	(−0.004, 0.006)	0.3	(−1.0, 1.8)
Work	0.001	(−0.001, 0.003)	0.2	(−0.4, 0.9)
Total	0.140	(0.061, 0.219)	38.1	(16.1, 68.5)
Unmediated	0.228	(0.088, 0.368)	61.9	(31.5, 83.9)
Total	0.368	(0.249, 0.487)	100.0	
Parents-in-law (*n* = 10,887)				
Mediated by:				
Caregiving	0.162	(0.085, 0.239)	44.0	(22.2, 75.4)
Coresidence	0.001	(−0.002, 0.004)	0.1	(−0.4, 1.0)
Work	0.005	(−0.000, 0.011)	1.0	(−0.1, 2.5)
Total	0.210	(0.113, 0.309)	45.1	(23.1, 76.7)
Unmediated	0.255	(0.085, 0.428)	54.9	(23.3, 76.9)
Total	0.465	(0.323, 0.608)	100.0	

^a^Adjusted for age, educational attainment, having a spouse, household expenditure, coresidence, and work in the previous year in all models

^b^Bootstrap-estimated with 3000 replications

## Discussion

We examined how OPNC affects the lifestyle and psychological distress of middle-aged women, using the data obtained from a nationwide longitudinal survey in Japan. Unlike most previous studies, we examined the impact of OPNC on caregiving, coresidence, and work, adjusted for their potential interactions and the effects from their previous statuses.

Results confirmed that about 30% of women began caregiving for their parents or parents-in-law in response to their need for care during the survey period (2019–2013). We also observed that the probability of becoming a caregiver was positively associated with previous coresidence with parents, a finding that was consistent with the result of a previous study conducted outside Japan [27]. Compared to the impact on the probability of becoming a caregiver, the probability of coresidence with parents was less sensitive to OPNC. In line with the results of previous studies [2, 4, 5] we obtained some evidence that OPNC prompted individuals to coreside with their parents, but the impact was rather small. Women who have been residing separately from parents seem to prefer going to their parents’ house to take care of them at least at the onset of the need for caregiving. The impact on work status was also limited, which was generally in line with the results of previous studies [6, 7].

Hence, we can argue that middle-aged women tend to respond to OPNC mainly by becoming a caregiver, at least initially, without substantial adjustments to coresidence with parents and work status. One possible explanation, which seems to be relevant in Japan, where intergenerational family setting is common, is that the parent-child coresidence, along with the wife’s labor force participation, may reflect the implicit contract regarding informal care and other life arrangements, which is traditionally made between adult children and their parents before OPNC [28, 29].

At the same time, results underscore the fact that OPNC is a stressful event for middle-aged women. OPNC raised psychological distress and its adverse impact was substantially mediated by becoming a caregiver. Coresiding with parents and work did not explain the variations in women’s psychological distress after including OPNC as an explanatory variable. This observation was consistent with the finding that women tended to become caregivers with limited adjustments to coresidence and work.

Additionally, the present study highlights that the kin relationship tends to confound the impact of caregiving on psychological distress. Compared to parents, the adverse impacts of both OPNC and caregiving on psychological distress were higher for parents-in-law. This observation confirmed the importance of kin relationship between caregivers and care recipients for a caregiver’s psychological distress, as already evidenced by previous studies [28–30].

We recognize that the present study has several limitations. First, we did not assess caregiving burden in terms of time spent on caregiving or the level of care required in the statistical analysis. This requires us to be cautious in any generalization of the obtained results. Second, we ignored the impact of prolonged caregiving on women’s lifestyle and psychological distress. As caregiving continues and the nursing care levels increase, women are more likely to adjust their lifestyle and feel more distressed, especially if the conflict between informal care and other roles becomes incompatible [20]. In this sense, it is likely that the present study may underestimate the impact of OPNC on women’s lifestyle and psychological distress. Following previous longitudinal studies (e.g., [31–33]), the dynamics of caregiving and its associations with lifestyle and mental health of caregivers, care recipients, and their family members must be addressed using more detailed longitudinal data. Third, we must expand the analysis to address how wider aspects of women’s multiple roles including interpersonal relations with others and other social ties are affected by OPNC [16, 19].

## Conclusions

Overall, the results highlighted that the onset of caregiving tends to be a serious external event that affects middle-aged women’s psychological distress, even if its impact on their lifestyle is relatively limited. If long-term care for the elderly keeps relying heavily on informal caregiving at home, policy measures to support informal caregivers are required. Providing a wider range of home-visit nursing care services to in-house care recipients and expanding institutional care services could be helpful in mitigating any psychological pressure and stress caused by informal caregiving at home.

## Abbreviations

K6: Kessler Psychological Distress Scale
MHLW: Ministry of Health, Labour and Welfare
OPNC: Occurrence of parents’ need for care
SUR: Seemingly unrelated regression

## References

[1] Organisation for Economic Co-operation and Development (OECD) (2011). Help wanted? Providing and paying for long-term care.

[2] Brown JW, Liang J, Krause N (2002). Transitions in living arrangements among elders in Japan: does health make a difference?. J Gerontol B Psychol Sci Soc Sci.

[3] Mizuno T, Takashaki K (2005). Caring for a yobiyose-rojin: a comparison of burden on daughters and daughters-in-law. J Gerontol Nurs.

[4] Takagi E, Silverstein M (2011). Purchasing piety? Coresidence of married children with their older parents in Japan. Demography.

[5] Takagi E, Silverstein M, Crimmins E (2007). Intergenerational coresidence of older adults in Japan: conditions for cultural plasticity. J Gerontol B Psychol Sci Soc Sci.

[6] Bauer J, Sousa-Poza A (2015). Impacts of informal caregiving on caregiver: employment, health and family. J Popul Ageing.

[7] Lilly MB, Laporte A, Coyte PC (2007). Labor market work and home care’s unpaid caregivers: a systematic review of labor force participation rates, predictors of labor market withdrawal, and hours of work. Milbank Q.

[8] He D, McHenry P (2016). Does formal employment reduce informal caregiving?. Health Econ.

[9] MacKinnon DP (2008). Introduction to statistical mediation analysis.

[10] Mackinnon DP, Dwyer JH (1993). Estimating mediated effects in prevention studies. Eval Rev.

[11] Cooper C, Balamurali TB, Livingston G (2007). A systematic review of the prevalence and covariates of anxiety in caregivers of people with dementia. Intl Psychogeriatr.

[12] Ennis E, Bunting BP (2013). Family burden, family health and personal mental health. BMC Public Health.

[13] Pinquart M, Sörensen S (2003). Differences between caregivers and noncaregivers in psychological health and physical health: a meta-analysis. Psychol Aging.

[14] Ho SC, Chan A, Woo J (2009). Impact of caregiving on health and quality of life: a comparative population-based study of caregivers for elderly persons and noncaregivers. J Gerontol A Biol Sci Med Sci.

[15] Legg L, Weir CJ, Langhorne P (2012). Is informal caregiving independently associated with poor health? A population-based study. J Epidemiol Community Health.

[16] Cannuscio CC, Colditz GA, Rimm EB (2004). Employment status, social ties, and caregivers’ mental health. Soc Sci Med.

[17] Litwin H, Stoeckel KJ, Roll A (2014). Relationship status and depressive symptoms among older coresident caregivers. Aging Ment Health.

[18] Marks N, Lambert JD, Choi H (2002). Transitions to caregiving, gender, and psychological well-being: a prospective U.S. national study. J Marriage Fam.

[19] Robison J, Fortinsky R, Kleppinger A (2009). A broader view of family caregiving: effects of caregiving and caregiver conditions on depressive symptoms, health, work and social isolation. J Gerontol B Psychol Sci Soc Sci.

[20] Stephens MAP, Townsend AL, Martire LM (2001). Balancing parent care with other roles: interrole conflict of adult daughter caregivers. J Gerontol B Psychol Sci Soc Sci.

[21] Kessler RC, Andrews G, Colpe LJ (2002). Short screening scales to monitor population prevalences and trends in non-specific psychological distress. Psychol Med.

[22] Kessler RC, Green JG, Gruber MJ (2010). Screening for serious mental illness in the general population with the K6 screening scale: results from the WHO world mental health (WMH) survey initiative. Int J Methods Psychiatr Res.

[23] Furukawa TA, Kawakami N, Saitoh M (2008). The performance of the Japanese version of the K6 and K10 in the world mental health survey Japan. Int J Methods Psychiatr Res.

[24] Sakurai K, Nishi A, Kondo K (2011). Screening performance of K6/K10 and other screening instruments for mood and anxiety disorders in Japan. Psychiatry Clin Neurosci.

[25] Organisation for Economic Co-operation and Development (OECD) (2015). In it together: why less inequality benefits.

[26] Greene WH (2012). Econometric analysis.

[27] Pezzin LE, Pollak RA, Schone BS (2014). Bargaining power, parental caregiving, and intergenerational coresidence. J Gerontol B Psychol Sci Soc Sci.

[28] Kurasawa S, Yoshimasu K, Washio M (2012). Factors influencing caregivers’ burden among family caregivers and institutionalization of in-home elderly people cared for by family caregivers. Environ Health Prev Med.

[29] Sugihara Y, Sugisawa H, Nakatani Y, et al. Longitudinal changes in the well-being of Japanese caregivers: variations across kin relationships. J Gerontol B Psychol Sci Soc Sci 2004; 59: P177–P184. 10.1093/geronb/59.4.p177 15294921

[30] Pinquart M, Sörensen S (2011). Spouses, adult children, and children-in-law as caregivers of older adults: a meta-analytic comparison. Psychol Aging.

[31] Amirkhanyan AA, Wolf DA (2006). Parent care and the stress process: findings from panel data. J Gerontol B Psychol Sci Soc Sci.

[32] Bookwala J (2009). The impact of parent care on marital quality and well-being in adult daughters and sons. J Gerontol B Psychol Sci Soc Sci.

[33] Smith GR, Williamson GM, Miller LS (2011). Depression and quality of informal care: a longitudinal investigation of caregiving stressors. Psychol Aging.

